# A review of recent studies on toxicity, sequestration, and degradation of per- and polyfluoroalkyl substances (PFAS)

**DOI:** 10.1016/j.jhazmat.2022.129120

**Published:** 2022-05-11

**Authors:** Rebecca A. Dickman, Diana S. Aga

**Affiliations:** Department of Chemistry, The State University of New York at Buffalo, Buffalo, NY 14260, United States

**Keywords:** Per- and polyfluoroalkyl substances (PFAS), Toxicity, Remediation, Decarboxyation, defluorination

## Abstract

The fate, effects, and treatment of per- and polyfluoroalkyl substances (PFAS), an anthropogenic class of chemicals used in industrial and commercial production, are topics of great interest in recent research and news cycles. This interest stems from the ubiquity of PFAS in the global environment as well as their significant toxicological effects in humans and wildlife. Research on toxicity, sequestration, removal, and degradation of PFAS has grown rapidly, leading to a flood of valuable knowledge that can get swamped out in the perpetual rise in the number of publications. Selected papers from the *Journal of Hazardous Materials* between January 2018 and May 2022 on the toxicity, sequestration, and degradation of PFAS are reviewed in this article and made available as open-access publications for one year, in order to facilitate the distribution of critical knowledge surrounding PFAS. This review discusses routes of toxicity as observed in mammalian and cellular models, and the observed human health effects in exposed communities. Studies that evaluate of toxicity through *in-silico* approaches are highlighted in this paper. Removal of PFAS through modified carbon sorbents, nanoparticles, and anion exchange materials are discussed while comparing treatment efficiencies for different classes of PFAS. Finally, various biotic and abiotic degradation techniques, and the pathways and mechanisms involved are reviewed to provide a better understanding on the removal efficiencies and cost effectiveness of existing treatment strategies.

## GRAPHICAL ABSTRACT

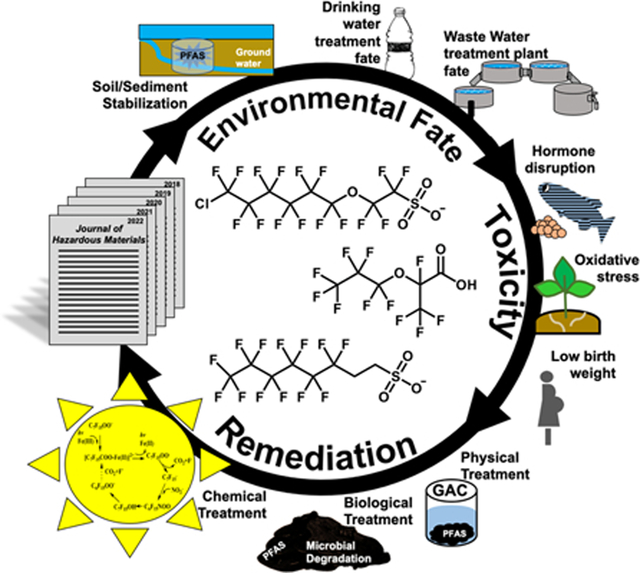

## 1. Introduction

Per- and polyfluoroalkyl substances (PFAS) are chemicals that have been widely used in commercial and industrial products since the 1940^′^s. Referred to as “forever chemicals”, PFAS are composed of a fluorinated alkyl chain and a polar head group that give them surfactant-like properties; PFAS can withstand extreme temperatures and are resistant to water and grease. These chemicals can enter the environment through a number of sources, including point source pollution from a production plant (Davis et al., 2007), effluent from an industrial waste water treatment facility (Ahrens et al., 2011; Hu et al., 2016), aqueous firefighting foams (AFFF) used at military sites (Houtz et al., 2013; Barzen-Hanson et al., 2017) and landfill leachates (Busch et al., 2010). Studies published over the past 30 years have identified PFAS in various environmental compartments such as surface water, rain water, drinking water, (Hu et al., 2016; Guardian et al., 2020; Sun et al., 2016; Shoemaker et al., 2008; Rahman et al., 2014; Quiñones and Snyder, 2009; Ingelido et al., 2018; Gobelius et al., 2019; Banzhaf et al., 2017) and groundwater, (Houtz et al., 2013; Barzen-Hanson et al., 2017; Xu et al., 2021; Kaserzon et al., 2019; Backe et al., 2013) and in manufactured goods like cosmetics, (Mousavi et al., 2021; Vestergren et al., 2013) food packaging, (Zabaleta et al., 2016; Trier et al., 2011) as well as agricultural foodstuffs. Vestergren et al. (2013); Sepulvado et al. (2011); Blaine et al. (2014); Blaine et al. (2013); Blaine et al. (2012); Genualdi et al. (2017) The physico-chemical properties of PFAS results in very low potential for excretion, which leads to bioaccumulation and biomagnification in humans (Zhou et al., 2021; Xu et al., 2019; Siebenaler et al., 2017; Carrizosa et al., 2021) and wildlife. Vestergren et al. (2013); Zhao et al. (2013); Xia et al. (2013); Miranda et al. (2021); Martin et al. (2003); Hong et al. (2015); Haukas et al. (2007) Their ubiquity and persistence, in addition to the significant negative health effects elicited by PFAS, (Ojo et al., 2021; Fenton et al., 2021; Danish Environmental Protection Agency Report, 2015) warrant the need to monitor these chemicals in drinking water, food products, and other environmental compartments.

Analysis of PFAS is typically performed by a targeted quantitative approach using liquid chromatography with tandem mass spectrometry (LC/MS/MS). The targeted techniques are robust for the analytes optimized in the method, but are limited to the handful of PFAS for which reference standards are available. Recently, suspect screenings and not-target analyses (NTA) have become common approaches as they provide an opportunity to increase the coverage of PFAS identification and improve our knowledge of their distribution in the samples of interest. Novel techniques like COnductor like Screening MOdel for Real Solvents (COSMO-RS) (Guardian et al., 2021) and in-silico PFAS fragmentation modeling, (Getzinger and Ferguson, 2021) combined with data mining software (Koelmel et al., 2020) have been used recently to identify emerging PFAS and resolve their isomers in environmental samples.

**Fig. 1.**
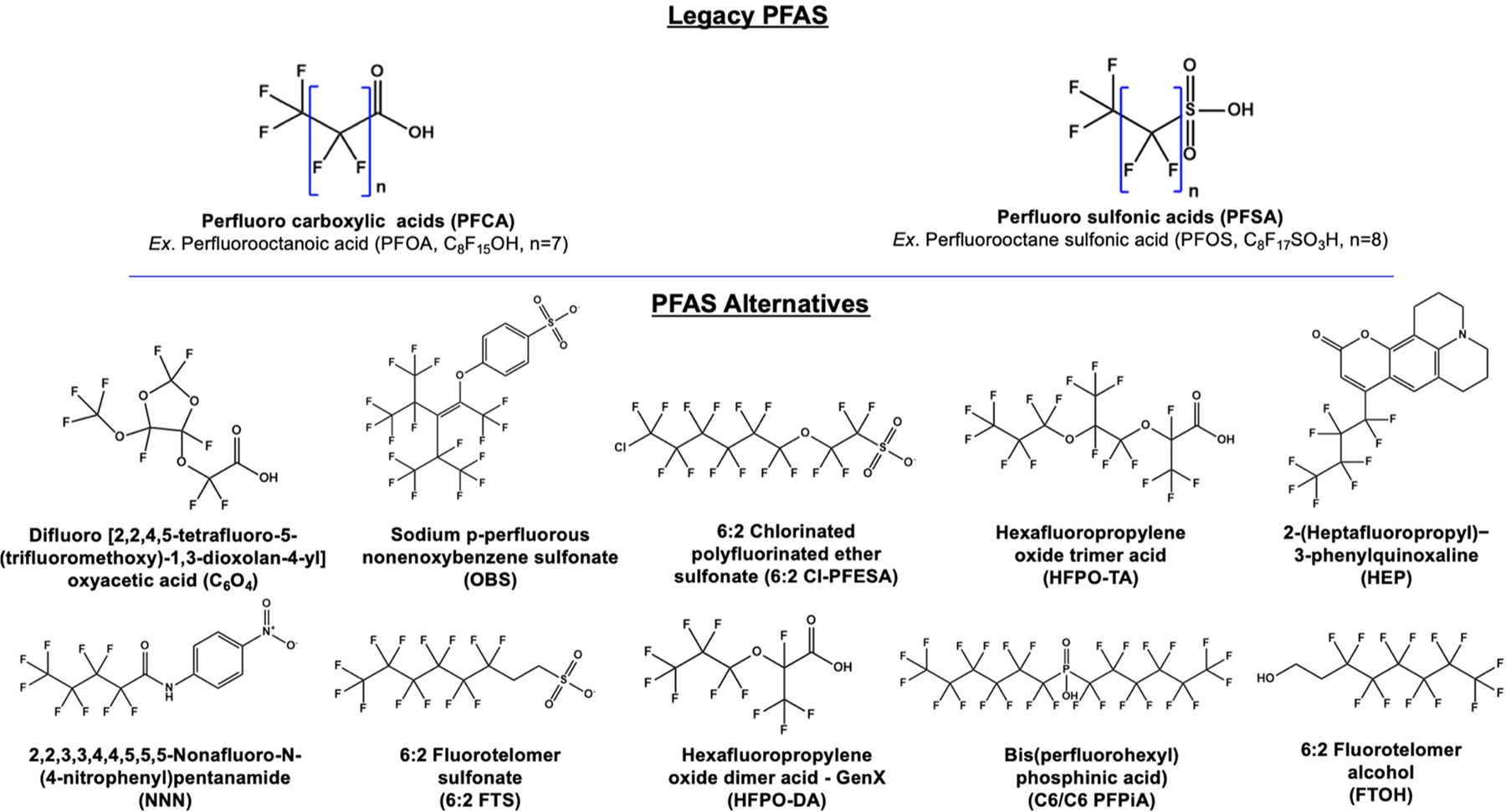
Examples of legacy and alternative PFAS compounds and their structures. The alternative PFAS discussed in this review are displayed in order of their mention in the text. Numerous legacy and alternative PFAS are not identified in this list, but are recognized as equally important substance for environmental monitoring and remediation.

PFAS can elicit significant negative health effects on humans and wildlife. Epidemiological studies have evaluated health impacts on populations that have been unintentionally exposed to PFAS, with findings linking various cancers, elevated cholesterol levels, decreased immune and liver functionalities, and significant birth defects. Bone-feld-Jorgensen et al. (2014) The discovery of these health concerns has led to the production phase out of long chain PFAS under the Stockholm convention. Following the phase-out, novel PFAS substitutes were produced, which included structural modifications such that PFAS are more easily excretable and/or degradable. These PFAS alternatives consist of shorter fluoroalkyl chains, and structures with ether linkages or chlorinated carbons (instead of having all fluorinated carbons) (Fig. 1).

Environmental remediation of PFAS-contaminated sites has been pursued using physical, chemical, and biological treatment strategies. Physical treatment is defined here as an approach that focuses on the isolation and removal of PFAS contamination, which often includes anion exchanger or carbon-based sorption materials. However, removal of the contaminant alone is not sufficient for complete remediation, as it only transfers PFAS from one medium, and concentrates them into a new medium; it does not necessarily degrade or destroy these contaminants. Chemical treatment is defined here as a remediation technique where controlled chemical pathways, including electrochemical, photochemical, and plasma reactions are applied to effectively degrade PFAS. Biological treatment, or biodegradation, refers to the degradation of PFAS by living organisms such as microbes and plants. Pairing physical removal with chemical or biological degradation treatments can reduce costs and efforts for remediation; some synergistic studies that incorporate biological and chemical treatments will be discussed in this review. Additionally, recent publications on the fate of PFAS in the environment have provided new information on what happens to these compounds in natural systems. Understanding the transport and transformation of PFAS in a contaminated environment can aid in the development of efficient bioremediation efforts, as well as improve chemical treatment technologies for PFAS remediation.

Given the widespread occurrence of PFAS, we must advance our understanding of their toxicity, especially as mixtures, in order to provide scientific basis for their regulation in consumer goods and in the environment by government agencies. Furthermore, effective remediation techniques that will completely eliminate legacy, novel, and transformation by-products of PFAS in drinking water sources are imperative. By highlighting 59 papers published in the *Journal of Hazardous Materials*, this review aims to compile and disseminate information on these three important topics: (1) ecological, animal, and human PFAS toxicity, (2) physical, chemical, and biological treatment for the removal and degradation of PFAS, and, (3) environmental fate of legacy and emerging PFAS. The papers cited in this review, dating from January 2018 to May 2022, are made *open-access* for one year following the publication of this review article. The goal of this review is to highlight significant findings on PFAS by researchers who published their papers in the *Journal of Hazardous Materials* and provide up-to-date information to the greater scientific community on the direction and advances on PFAS research as a whole.

## 2. Toxicity of PFAS

The toxicity (Bonefeld-Jorgensen et al., 2014; Lau, 2015; Lau et al., 2004, 2006; Steenland et al., 2020) and bioaccumulation (Conder et al., 2008; Kelly et al., 2009) of PFAS have been widely studied to date, however, toxicity of PFAS mixtures as they appear in the environment is still poorly understood. The most widely studied PFAS are the perfluorooctanoic acid (PFOA) and perfluorooctane sulfonic acid (PFOS) (Fig. 1) (DeWitt et al., 2009; Gordon, 2011; Cui et al., 2008; Trudel et al., 2008) because they are the most used forms and are stable end products of other PFAS precursors. Martin et al. (2010); Zhao et al. (2018) New studies on the toxicity of PFOA and PFOS (also referred as “legacy PFAS”), along with replacement PFAS (also referred to as “alternative PFAS”) based on animal and cell studies, epidemiological studies in humans, and computational models, continue to display the endocrine disruption and toxicity of PFAS.

### 2.1. Ecological toxicity

Effects of PFAS contamination on plant and animal health has been widely evaluated in locations where point source pollution is a known concern. It has been shown that PFOA exposure results in decreased shoot growth for *V. radiata* and *O. sativa* when grown in PFOA contaminated soil (100, 300 mg / kg dry soil respectively). Kwak et al. (2020) Decreased seedling sizes in contaminated soils was also observed in this study. Algal soil biomasses of *C. infusionum* and *C. reinhardtii* were significantly reduced in 50 and 700 mg / kg dry soil, respectively. Nematode reproduction rates decreased after only 1 day of exposure to PFOA contaminated soils at or above 300 mg / kg dry soil, compared to those in control soil. For earthworms, PFOA contamination above 600 mg / kg dry soil decreased survival rate compared to the control (no PFOA). Kwak et al. (2020) Plant uptake and phytotoxicity of PFOA and PFOS (500 and 5000 ng/L) were evaluated in lettuce exposed to the contaminants over a 28-day period. Li et al. (2020) The 28-day exposure provided no evidence of phenotype or biomass changes. The accumulation of PFOA was measured to be 4.0 – 4.3 times higher than PFOS. Based on a metabolomics study approach, significant changes in the carbon energy metabolome were observed as a result of the PFOA and PFOS contamination at both 500 and 5000 ng/L exposure. Carbon metabolism was affected by both contaminants in the citric acid cycle, pyruvate, glyoxylate, and decarboyxlate pathways. Additionally, PFOA exposure was observed to induce stress for the amino acid metabolome, while PFOS exposure disrupted linoleic acid metabolism. Four major plant defense pathways were triggered as a result of environmental stress of PFOA and PFOS exposure. A biomarker for oxidative DNA damage, 8-Hydroxy-deoxyguanosine, was also observed to be upregulated by PFOA and PFOS exposure, with a 2.0–4.1-fold increase in comparison to the control. The metabolomics workflow used in this study provided numerous significant examples of the impacts of PFAS contamination on plants, which would not have been readily observed if the study only evaluated changes on biomass or phenotype alone. However, effects of PFAS on lettuce growth could not be observed at the low levels of exposure performed in this study. Li et al. (2020).

The distributions of PFOS in overlaying water, pore water, suspended particulate matter, sediment, and three benthic organisms; *H. azteca, L. hoffmeisteri, and C. kiiensis*, were studied after incubation with PFOS contaminated soil. Wu et al. (2021) The accumulation of PFOS in the benthic organisms was < 2% of the overall mass balance; the majority (66–80%) remained in the sediment. *C. kiiensis’* bioturbation had no effect on the redistribution of PFOS while *H. azteca* and *L. hoffmeisteri*, promoted the release of contaminant from the sediment. *H. azteca* and *L. hoffmeisteri* also resulted in the mortality of the *C. kiiensis* by destruction of its habitat, and increased exposure to PFOS released from the sediment. This study provides evidence that benthic organisms, like *H. azteca* and *L. hoffmeisteri*, can affect the bioavailability of PFAS within soils, which in turn impacts other biota within the environment. Wu et al. (2021).

### 2.2. Animal toxicity

In addition to environmental models, the effects of PFAS on wildlife have been evaluated. Metabolomics was used in a study on *Ruditapes philippinarum* clams to compare PFOA and the PFAS alternative, difluoro [2,2,4,5-tetrafluoro-5-(trifluoromethoxy)− 1,3-dioxolan-4-yl]oxyacetic acid (C_6_O_4_), on the marine species. Fabrello et al. (2021) Haemocyte cell count and size decreased in the presence of both contaminants. The alternative PFAS lead to biomarker fluctuations for haemocytes and gills, as well as caused more DNA modifications and damage than PFOA, at lower concentrations. Fabrello et al. (2021) The impacts of two other PFAS alternatives, 6:2 chlorinated polyfluorinated ether sulfonate (6:2 Cl-PFESA) and sodium p-perfluorous nonenoxybenzene sulfonate (OBS) (Fig. 1), were compared to that of PFOS in zebrafish embryos at a concentration of 1 μM. Huang et al. (2022) PFOS exposure was observed to have a more prominent impact on oxidative liver stress in zebrafish relative to 6:2 Cl-PFESA and OBS exposure. However, the trends in observed disruptions in gut microbiota, anti-inflammatory, as well as upregulation of immune-related gene expression were consistent between all three PFAS studied. Huang et al. (2022).

Effects of PFOA and PFOS on reproductive traits in Japanese Medaka were evaluated at concentrations of 10 mg/L and 1 mg/L respectively, for up to 21 days. Kang et al. (2019) Both PFOS and PFOA caused reproductive toxicity including modifications of gene and peptide levels, as well as changes in egg production. PFOA was observed to cause significant estrogenic effects while PFOS induced similar pathways of other anti-estrogenic compounds. It was observed that the PFOA and PFOS induced different effects on the Medaka, where the impacts from PFOA were consistent with the impacts from estradiol (E2), the estrogenic control, while PFOS exposure effects resembled the effects of prochloraz, the anti-estrogenic control. Upregulation in vitellogenin levels in male fish and reduced egg production in female fish are common manifestations of endocrine disruption caused by contaminants. These conclusions were drawn based on transcriptional changes in vitellogenin and other estradiol induced genes. These preliminary toxicity studies provide insights into how phenotype is effected by PFAS exposure. Future research on marine organisms can be used to inform future human toxicity studies, as the model organism can elucidate the impacts the contaminant has on phenotype and in this study, reproductive health. This model organism and toxicity workflow can be used to evaluate PFAS alternatives in addition to the common perfluoroalkyl sulfonic acids (PFSA) and carboxylic acids (PFCA). Kang et al. (2019).

**Fig. 2.**
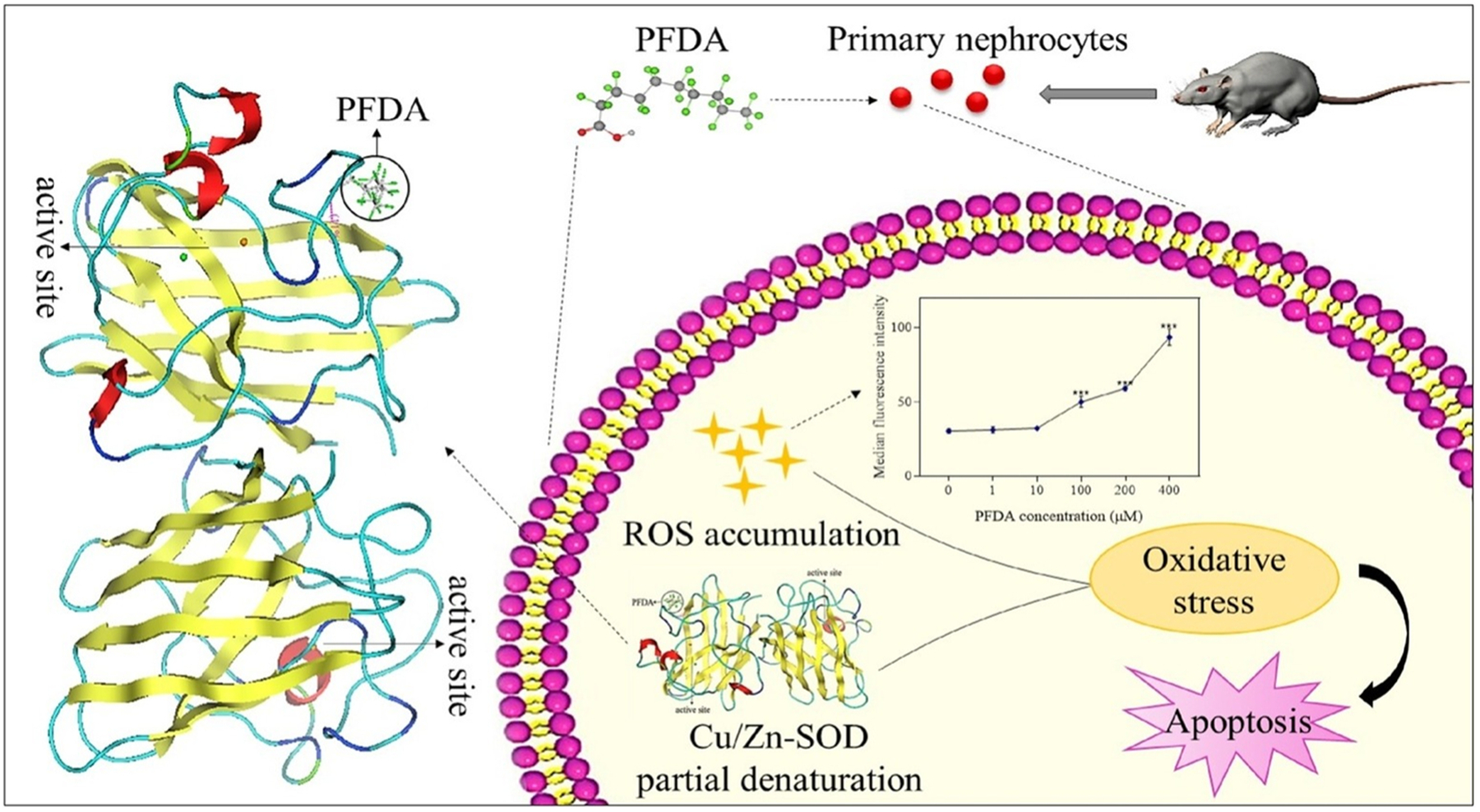
Gao et al., 2020. Induced oxidative stress in nephrocytes from PFDA exposure in mice. Exposure caused increased reactive oxygen species within cells as well as denaturation and inhibition of enzyme superoxide dismutase. This oxidative stress increased apoptosis in nephrocytes.

Early mice and rat PFAS exposure studies observed significant impacts on the liver, kidney, spleen as well as other organs. Gao et al. (2020); Guo et al. (2021); Jin et al. (2020); Zhou et al. (2018); Chen et al. (2021) Jin et al. observed that the precursor class of PFAS, perfluoroalkane sulfonyl fluorides (PFASFs), undergo reduction to form the more stable PFSAs while oxidizing both glutathione (GSH) and a mixture of glutathione and a cysteine containing peptide (PEP-S-SG) when inoculated into rat livers. Gao et al. (2020) The transformation of PFASFs to the more stable PFSAs is well documented, therefore understanding the side effects of the reduction on organisms is crucial. These findings imply that PFASFs can inhibit protein activity. Perera et al. (2022) Oxidative stress was also observed in mouse nephrocytes in the presence of perfluorodecanoic acid (PFDA) at both the cellular and molecular levels. Gao et al. (2020) Increased reactive oxygen species (ROS) at the cellular level and inhibition of superoxide dismutase lead to nephrocyte apoptosis since cells could not prevent or repair oxidative damages. The hydrogen bonding effects of PFDA on Cu/Zn-SOD lead to morphological changes in the enzyme structure and caused decreased enzymatic activity, which induced further oxidative stress (Fig. 2). Guo et al. (2021) A similar study reported decreased splenic weights in PFOA treated mice, as well as decreased splenic and serum iron levels. Evidence of anemia and macrophage levels consistent with an autoimmune disorder were observed, which supports the conclusion that upregulation of macrophage levels is a major pathway to splenic atrophy due to PFOA contamination. Guo et al. (2021) Male mice were inoculated with ether carboxylate PFAS, perfluoro-3,5,7,9-butaoxadecanoic acid (PFO4DA) and perfluoro-3,5,7,9,11-pentaoxadodecanoic acid (PFO5-DoDA), which caused weight gain and liver enlargement, as well as suppression of genes in both metabolic and stress pathways. Chen et al. (2021) The high potential of these compounds to bioaccumulate suggests that the ether PFAS (alternatives) are equally concerning contaminants as they also exhibit toxicity. In another study, male mice were inoculated with the PFOS alternative, 9-chlorohexadecafluoro-3-oxano-nane-1-sulfonate also referred to as 6:2 chlorinated polyfluorinated ether sulfonate (6:2 Cl-PFESA) (Fig. 1), which led to decreased testicular and epididymis weights. Zhou et al. (2018) Unlike PFOS, no significant impacts on testosterone, steroidogenic related genes or sperm count was observed with this PFAS alternative. Zhou et al. (2018) Another PFAS alternative, hexafluoropropylene oxide trimer acid (HFPO-TA), was evaluated for its effects on mitochondrial function and biogenesis in male mice livers. Xie et al. (2022) Several studies including metabolomics, genomics, enzyme linked immunoassay (ELISA), histological analysis, and transmission electron microscopy were preformed following the 6-week mouse liver exposure to HFPO-TA spiked drinking water (2, 20, and 200 μg/L). It was observed that liver weight, cell morphology, and inflammatory response were all impacted by the HFPO-TA exposure. Metabolites as well as mitochondrial DNA and RNA related to mitochondrial fusion and division were observed to significantly increase after high level (200 μg/L) exposure to HFPO-TA. HFPO-TA is used in place of PFOA, and has been observed in surface waters, which is concerning since this study indicates that HFPO-TA can significantly impact liver function and potentially induce liver cancer. Xie et al. (2022) These studies in animal models provide valuable insight into the mechanistic pathways of PFAS toxicity, and can be used to inform studies with human subjects.

### 2.3. Human toxicity

Because the toxicological effects of environmental contaminants on humans cannot be studied by exposing human subjects to the contaminant, the information on human toxicity is inferred from epidemiological, computational (*in silico*), *in vitro*, and *in vivo* studies. Computational modeling is a common technique that is used to understand contaminant risks and toxicity in order to minimize invasive sampling of human subjects. The adsorption, transport, and residence time of PFOS and two novel PFAS alternatives, OBS and 6:2 Cl-PFESA (Fig. 1), in the phospholipid bilayer were evaluated using molecular dynamics simulations. Lv and Sun (2021) Molecular dynamics simulations can be used to predict the interactions of PFAS with these biomolecules in full atomic detail and with very fine temporal resolution. The calculated energy barriers for the three compounds into the bilayer was low, suggesting that these compounds can seamlessly enter the bilayer of the cell membrane. Once inside the bilayer, interactions between the sulfonate head group and cationic N-atoms in PFAS lead to the restriction of the bilayer head group and changed the orientation of the bilayer. The effects that the head group had on the bilayer’s orientation were more pronounced for the two PFAS alternatives relative to PFOS. It was also found that with the incorporation of PFAS into the bilayer, the area per lipid in the bilayer decreased. However, PFAS has similar impacts on cholesterol that cause the bilayer to contract laterally, which leads to a widening of the bilayer. Lv and Sun (2021) *In-silico* analysis and machine learning approaches were also used to model the interactions of over 5, 000 PFAS with the human androgen receptors (HAR), and reported that 23 of the tested PFAS strongly interacted with HAR. Tachachartvanich et al. (2022) The results of these computational studies were then experimentally validated and the mechanisms of interaction were explored using triple negative human breast cancer cell line (MDA-kb2) and human prostate cancer cell line (VCaP) using the PFAS standards that were commercially available. This study confirmed that three PFAS alternatives, (9-(nonafluorobutyl)− 2,3,6,7-tetrahydro-1 H,5 H,11 H-pyrano[2,3-*f*]pyrido[3,2,1-ij]quinolin-11-one (NON), 2-(heptafluoropropyl)− 3-phenylquinoxaline (HEP), and 2,2,3, 3,4,4,5,5,5-nonafluoro-N-(4-nitrophenyl)pentanamide (NNN) (Fig. 1), had significant effects on HAR at environmentally relevant concentrations. It was observed that through competitive binding, HAR transactivation was inhibited by the PFAS and caused upregulation of HAR. Decreased expression of androgen regulated genes, PSA and FKBP5, signified antiandrogenic effects as a result of exposure. The results of the PFAS exposure studies were consistent with the positive control, hydroxyflutamide, a known AR inhibitor. Notably, it was found that the alternative PFAS had more prominent androgenic effects than what has been reported for their legacy PFAS counterparts. These results indicate that the *in-silico* model was sufficient in predicting the effects of endocrine disrupting chemicals. Tachachartvanich et al. (2022).

The comprehensive analysis of the changes in metabolome of living organisms in response to various stressors and their relationship to the organismal phenotype has been used to asses toxicity of contaminants. This metabolomics approach focuses on the analysis of primary and secondary metabolites that are of prime importance to obtain a biochemical perspective and a phenotypic fingerprint of organisms in their environment. A metabolomics analysis of lung, liver, and intestinal cells was preformed following exposure to PFOA. In this study, 67 metabolites in the A549 lung cells, 12 DLD-1 intestine metabolites and 10 liver metabolites were disrupted by exposure to PFOA. Zhang et al. (2021) Most metabolites affected were related to inflammation and cell cycle pathways, but the upregulation of vitamin B6 and arginine in A549 lung cells lead to decreased inflammatory effects. Zhang et al. (2021) Authors suggest that observed beneficial effects of B6 and arginine in the inflammatory response of A549 lung cells can be used to help resolve the negative impacts of PFOA exposure in humans. Metabolomics and proteomics studies were used to determine effects on Leydig cells treated with low dosages of PFOA (0, 0.1, 1 and 10 μM, 48 h). Huang et al. (2021) The proteins and metabolites affected in the PFOA-treated cells were associated with fatty acid metabolism and promotion of testicular steroidogenesis. The mechanism of PFOA toxicity on male reproductive hormones was corroborated by the results of the rat study as well, but should continue to be verified in human subjects. Huang et al. (2021).

Another study investigated the impact of PFAS on the Nrf2-ARE pathway for oxidative and toxicant stressors in ARE reporter-HepG2 cells. Ojo et al. (2022) The length of the alkyl chain had a direct relationship to the promotion of the Nrf2-ARE pathway, with perfluorodecanoic acid (PFDA) having the highest induction effect. The experimental toxicities were typically lower than the predicted values based on a concentration addition model, with the exception of the PFOA and PFOS mixture. Additionally, Sulfonated PFAS were observed to have synergistic effects when in mixtures, while carboxylated PFAS showed a clear additive trend that possibly lead to some of the deviation in the model. Ojo et al. (2022).

The interaction of human serum albumin (HSA) with PFAS alternative, GenX (Fig. 1), was evaluated using fluorescence and circular di-chroism titration experiments. Perera et al. (2022) GenX was observed to bind to HSA at multiple sites, and was observed to prefer the binding site closest to amino acid residue, Trp – 214. With the addition of cyclodextrins, β-CD and NH2-β-CD, it was observed that through host-guest encapsulation, GenX was extracted from the HSA binding site. With the addition of β-CD or NH2-β-CD, the fluorescence emissions returned to the level observed with HSA prior to GenX exposure. Perera et al. (2022) This study provides groundwork for the potential use of cyclodextrins for therapeutic treatment to reverse the binding of PFAS with proteins.

Three human epidemiological studies on PFAS toxicity were performed to evaluate effects of PFAS exposure on childbirth and early child development. The first study evaluated the weights of newborns and mothers in relation to prenatal exposure to PFAS. PFOA, PFOS, and 6:2 Cl-PFESA were found in all the mothers, with the highest concentrations observed in older, first time mothers. Xu et al. (2019) A positive correlation was observed between body mass index and both PFOS and 6:2 Cl-PFESA, which is a common correlation for other lipophilic contaminants. Xu et al. (2019) Additionally, birthweights and ponderal indices for affected newborns were lower in cases where prenatal exposure to PFOS occurred. Xu et al. (2019) This review highlights many of the toxicological effects of 6:2 Cl-PFESA, suggesting that this compound is important to monitor in the environment, but is currently not being widely included in targeted analyses. The mist suppressant, 6:2 Cl-PFESA, has been used as a PFAS substitute in electro-plating dating back to 1975, and has been recently detected in Chinese surface waters and metal plating workers. Wang et al. (2013).

6:2 Cl-PFESA, PFOS, PFOA, PFDA, PFNA, and PFHxA were also evaluated in neonate spinal cord sera (n = 942) from Wuhan, China. Liu et al. (2021) This study also monitored estrogen levels within the sera to determine the relationship between PFAS exposure and estrogen production in neonates. The results of this study showed that there was a strong correlation between the presence of PFAS and elevated levels of estrone (E1), estradiol (E2), and estriol (E3). This trend was observed for both individual PFAS as well as PFAS mixtures. The E2 levels were most prominently affected by 6:2 Cl-PFESA (48.5%) when assessed with a weighed quantile sum regression, while the acetic acid moieties (23.8–47.8%) studied had the largest impact on E3. Results from this study shows that PFAS can disrupt human hormone levels, but suggests that mechanistic studies are still needed to understand the impacts of PFAS on estrogenic hormones. Liu et al. (2021).

An evaluation of the developmental effects of PFAS on children up to 7 years of age from a cohort of 1240 mother child pairs was also performed. Carrizosa et al. (2021) PFOA and PFOS were detected in all maternal plasma samples, while PFHxS and PFNA were found in the majority of plasma samples (96, 99% respectively). Unlike most research on the subject, this study observed no significant impacts on mental development from children 14 months to 7 years old. Some association between PFAS exposure with memory and verbal skills, in children at 4–5 years of age, was observed, but it was not observed to be statistically significant. The author acknowledged that the contradicting reports in literature are enough to induce scrutiny to their results. Carrizosa et al. (2021) Contradicting results in epidemiological studies are not uncommon specially when the sample size is small. Therefore, un-avoidable interferences can lead to skewed results within epidemiological studies. With these recent findings on the toxicity of PFAS, the need to isolate and destroy these persistent environmental contaminants is warranted, and studies with larger cohorts are critical to perform.

## 3. Physical treatment

Research to develop technologies for PFAS remediation is a field with significant growth in the past decade; various physical, chemical, and biological treatment techniques will be reviewed below. The isolation and removal, by sorption or partitioning of PFAS from contaminated environmental compartments will be referred to as physical treatment. Technologies for efficient physical treatment of PFAS are needed, such as activated carbon and anion exchange sorbents.

### 3.1. Removal with carbon-based sorbents

Granular activated carbon (GAC) has been reported to effectively sorb common legacy PFAS, resulting in high removal efficiencies. Super fine powder activated carbon (SPAC) with a ceramic membrane filtration system was compared to traditional GAC for the remediation of ground water that is contaminated with AFFF. Murray et al. (2019) The very high surface area, as well as the increased meso to macro-pore ratio, gives the SPAC a higher sorption capacity than classic GAC. The SPAC showed a 480x improvement in the removal efficiency of AFFF contaminated waters than classic GAC; however, both sorbents showed decreasing mass loading capacities as the carbon chain length of PFAS decreased. Murray et al. (2019) GAC provides promise for PFAS removal in complex contaminated water samples, but the breakthrough of short chain (carbon chain <6) PFAS must be addressed in future research. The adsorption of long chain PFAS using GAC with aeration during drinking water treatment was evaluated in a lab-scale experiment, using environmentally relevant concentrations. Meng et al. (2020) Air pockets on the surface of the activated carbon enhanced adsorption of PFAS compounds (carbon chain > 7), suggesting that this technique could be a prime candidate for efficient drinking water PFAS treatment and should be evaluated at full-scale. Meng et al. (2020).

Similar to GAC, ordered mesoporous carbons (OMC) are carbon-based materials being investigated for PFAS removal from water in a batch adsorption experiment. Lei et al. (2021) The OMCs were calcinated at different temperatures (700, 900, 1100°C) and were characterized for the sorption of PFOA. Calcination is the process in which OMCs are purified and volatile compounds are removed from the material. The 700-OMCs had a lower adsorption capacity (29.6 mg/g per 1 h) for PFOA than the 900-OMC (82.9 mg/g per 1 h) due to the higher oxygen content available from lower temperature calcination. Lei et al. (2021) Oxygen-based functional groups on the surface of the OMC provides electrostatic repulsive effects on anionic PFOA. This causes the sorption of the 700-OMC to be poor, and not suitable for real-world applications. The study concluded that sorption of PFOA to the OMCs was through hydrophobic and electrostatic interactions, which is consistent with literature on carbon-based sorption materials. Kim et al. (2020); Lu et al. (2020) This study also reported that with the addition of humic acid (30 mg/L), a surrogate for natural organic matter, the removal of PFOA decreased (by roughly 25% in 900-OMC) due to competitive interactions. Lei et al. (2021).

### 3.2. Metal doped nanomaterial sorption

**Fig. 3.**
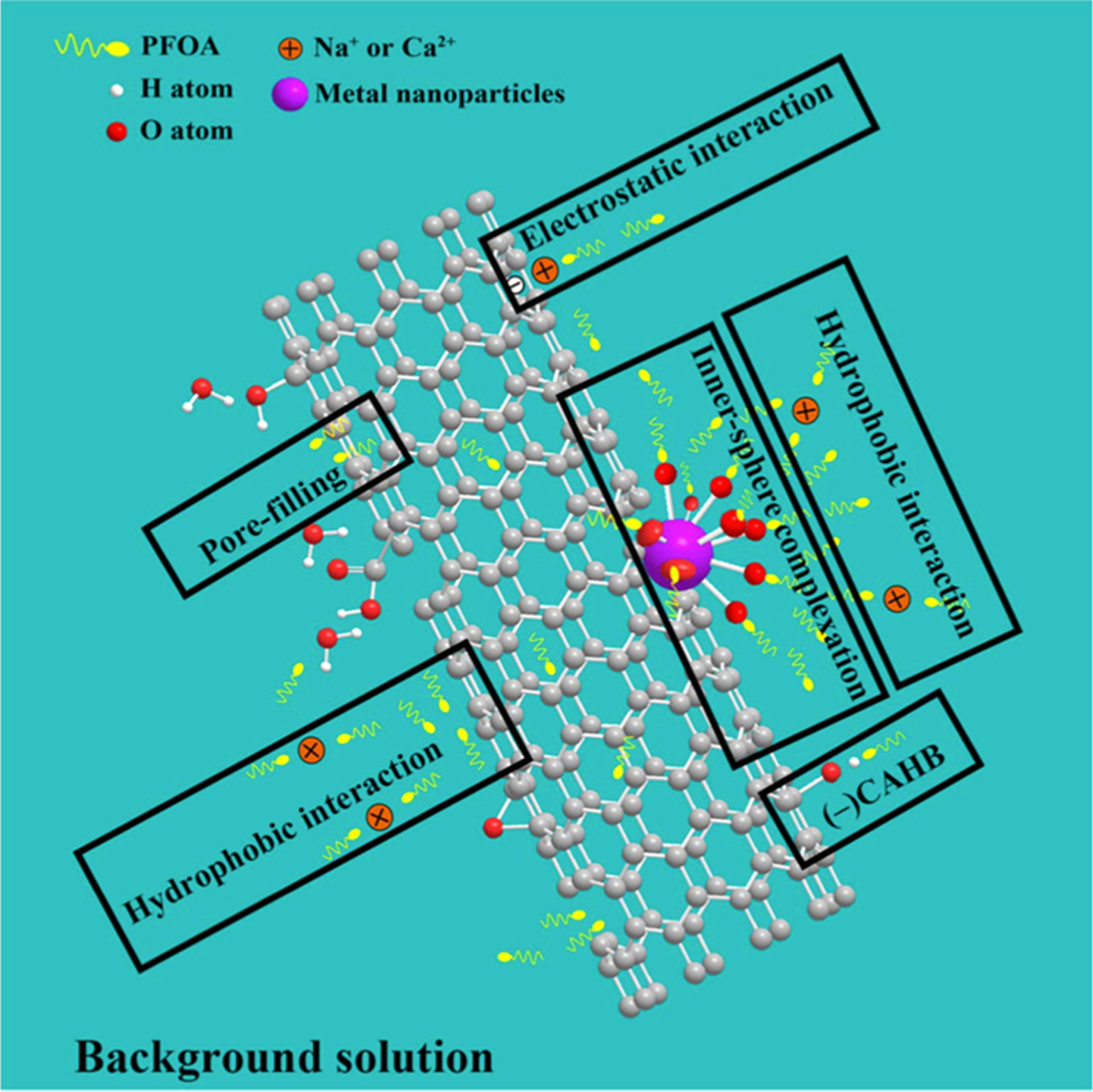
Liu et al.(2018) The diagrammatic sketch of main mechanisms about PFOA adsorbed on X/XOy-CNTs.

Carbon based nanoparticles have been evaluated for treatment of contaminants in the environment. For instance, multiwalled carbon nanotubules (MWCNT) doped with iron, copper, and zinc were evaluated for PFOA removal. Liu et al. (2018) Metal doped MWCNTs had higher removal efficiencies than the MWCNTs alone, especially when the metal was copper (17.99-fold > MWCNTs). Liu et al. (2018) Inner sphere complexing was a prominent interaction mechanism observed with the metal doped nanotubules in addition to the hydrophobic and electrostatic interactions, while carbon nanotubules alone only interacted through the latter (Fig. 3). The metal doped nanotubules were efficient in removing PFOA even at trace, environmentally relevant levels, making it a promising new direction for PFAS removal. Liu et al. (2018) This work was followed by a study on electro sorption of PFOA on reduced aerogel modified with copper nanoparticles and fluorine atoms (Cu/F-rGA). Liu et al. (2021) The Cu/F-rGA aerogel electrode showed significantly higher removal rate (489% more PFOA removal) than with an open circuit electrode alone. Due to its high removal efficiency and regeneration potential, the Cu/F-rGA aerogel is a great technology to further explore for PFAS removal, (Liu et al., 2021) however, these materials have not been tested for sorption of short chain and novel PFAS.

**Fig. 4.**
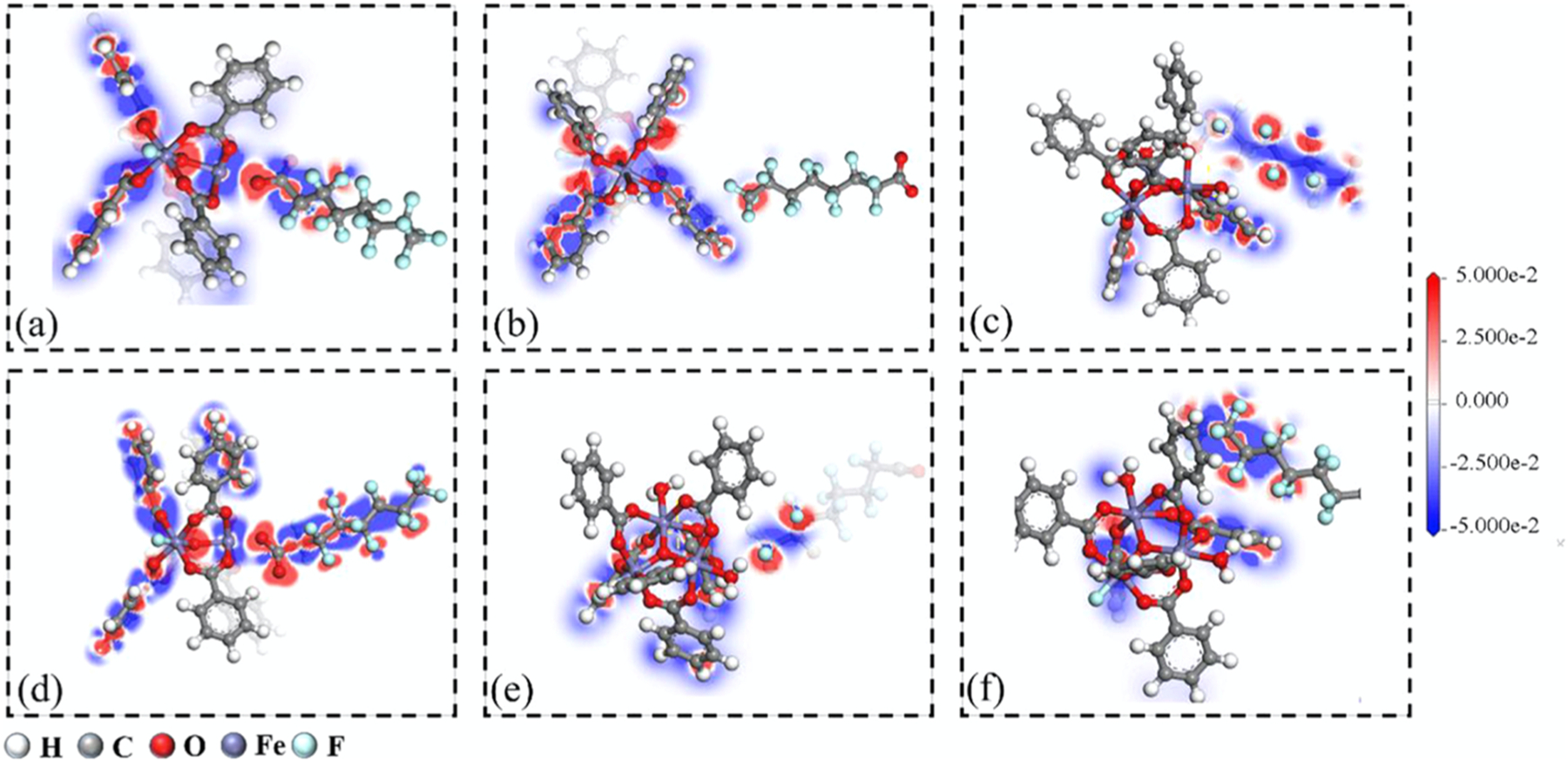
The differential charge images: (a) LAB1, Lewis acid/base complex between PFOA and Fe_3_O cluster; (b) π-CF_1_, C-F chain tail reacts vertically with benzene ring; (c) π-CF_2_, C-F chain tail reacts parallel with benzene ring; (d) LAB2, Lewis acid/base complex between PFOA and protonated Fe_3_O cluster; (e) π-CF_3_, C-F chain tail reacts vertically with benzene ring of protonated Fe_3_O cluster; (f) π-CF_4_, C-F chain tail reacts parallel with benzene ring of protonated Fe_3_O cluster.

Similar to metal doped nanoparticles, iron-based metal organic frameworks (MOFs) were characterized and used for PFOA removal. Yang et al. (2020) Adsorption through π-carbon-fluorine interactions, hydrogen bonding, anion-π interaction, but most prominently with Lewis acid/base complexing was observed (Fig. 4). The shape of the pore window played a role in adsorption, with the triangular and pentagonal structures having the highest capture. This study elucidated the interactions between PFAS and iron complexes within the MOF structure using density functional theory (DFT). The metal doped nanotubule study provided evidence that the metal intersphere interactions were stronger than the hydrophobic and electrostatic interactions within the system. An experimental comparison of the MOF to traditional carbon sorbent is still necessary to verify the DFT calculations. Additionally, evaluation of the efficiency of MOF’s for the sequestration for other PFAS is still needed, but the characterization of the MOFs in this study is a step in the right direction. Yang et al. (2020).

### 3.3. Hydrotalcite and anion exchange

Layered double hydroxide such as hydrotalcite, or anionic clays, have a sheet of positively charged surfaces providing them with strong anion exchange capacities. The calcined hydrotalcite was found to be effective for PFOA removal, due to its high anion exchange capacity and large specific surface area. Chang et al. (2019) PFOA adsorbed on the surface and intercalated into the crystal structure of the calcined hydrotalcite. Additionally, the sorption was found to be independent of pH and with a removal capacity of 1587 mg/g, suggesting that they can be used for environmental remediation efforts. Chang et al. (2019) Like many of these materials, the need for further evaluation on other PFAS compounds is necessary. A separate study evaluated a similar hydrotalcite material with a composition of Cu(II)Mg(II)Fe(III) for the sorption of PFBS, PFBA, PFOA and PFOS. Chen et al. (2021) The bottom up process for material synthesis produced very small particle sizes (d50 = 3 μm) which allowed for rapid adsorption of the PFAS contaminants (95% removal in 1 h). This study examined the relationship between sorption rates and carbon chain length, and concluded that hydrophobic interactions were the dominant mechanism relative to ion exchange interactions. Ion exchange interactions were still present with this material, as observed by the negative impact of alkaline conditions on sorption rates of PFOA. It was also observed in this study that the sulfonic acid moieties can outcompete other anionic contaminants, including the carboxylates. Chen et al. (2021) This is a common observation likely due to Van der Waals interactions, as sulfonated PFAS have more retention in a C18 liquid chromatography analyses in comparison to their carboxylated counterparts. United States Environmental Protection Agency (2021).

The removal efficiency of a commercial macroporous anion exchange resin, Purolite^®^ A860, was evaluated for a novel class of PFAS, ether carboxylates (*e.g*. GenX), in surface and recycled waste water. Dixit et al. (2020) The resin is acrylate based with quaternary ammonium cationic functional groups for anion exchange. It was found that this resin significantly removed PFAS ethers from a contaminated water, reducing concentrations from 50 μg/L to below the United States Environmental Protection Agency (USEPA) drinking water (70 ng/L lifetime (United States Environmental Protection Agency, 2016)) advisory in 20 min, while also removing natural organic matter and inorganic ions. Dixit et al. (2020) The results of the study suggest that this is a viable material to employ for drinking water treatment of PFAS.

### 3.4. Other removal techniques

Molecularly imprinted polymers (MIP) are carbon-based polymers etched with specific binding sites, similar to natural receptors such as enzymes. MIPs have been recently used to capture organic pollutants from environmental matrices due to the specific nature of the material. In this study, MIPs were synthesized to have two functional groups for use in PFOS sequestration. Guo et al. (2018) The MIPs were shown to have rapid and selective binding for PFOS, reaching equilibrium within 1-hour. The most effective condition (pH 3, ambient temperature) yielded a binding capacity of 75.99 mg PFOS per gram of MIP. The combined molecular pore size and electrostatic interactions dominated the sorption between PFOS and the MIPs. Guo et al. (2018) This technology should be tested with other PFAS to evaluate their general applicability. The regeneration potential of the MIPs for reuse in remediation of PFAS was demonstrated in this study. The reusability of the MIPs, in addition to the observed rapid binding potential, suggests that this is a good resource to explore for PFAS remediation.

Extraction of PFAS from aqueous media using liquid-liquid extraction is another approach to PFAS removal and remediation in the environment. Various additives for this process can assist the partitioning of PFAS into the organic layer. The use of an ionic liquid, methyltrioctylammonium bis(trifluoromethylsulfonyl)imide ([A336] [NTf2]) dissolved in n-octanol, was evaluated for the extraction of PFOA from water. Zhang et al. (2021) This extraction was pH, temperature, and concentration dependent, with a 55.6% removal of PFOA after 30 min. At concentrations of 20–500 mg/L, 0.65–88.21% of PFOA was removed from the aqueous phase suggesting that this technique is more suitable for high concentration contamination such as aqueous fire-fighting foams. The use of ionic liquids for extraction may be a sustainable practice for remediation, but the organic salt should first be considered for biodegradation. Additionally, before application in contaminated waste or ground waters, it must first be demonstrated that this material is effective atsorbingi PFAS at environmentally relevant concentrations (<20 mg/L). Zhang et al. (2021) Similarly, a cysteine based green surfactant was used to remove PFOA from water samples with foam fractionation. Ziaee et al. (2021) The surfactant 1-octanoyl-cysteine was effective in removing 72% of PFOA from water contaminated with 40 mg/L after 1 h. This surfactant is biodegradable, which suggest that 1-octanoylcysteine may be an effective green solution for remediation of PFAS contaminated waters. Ziaee et al. (2021) Both additives should be tested further to evaluate their effectiveness in assisting removal of novel PFAS, especially the more polar, short chain PFAS.

## 4. Chemical treatment

As discussed above, physical treatment is a valuable step for the remediation of PFAS contamination in source and drinking waters. But the removal of PFAS creates a secondary problem: an abundance of PFAS-contaminated materials that, in turn, require permanent disposal solutions. Disposal of these materials can be costly and challenging and inevitably lead to PFAS in leachate. Degradation of PFAS is therefore critical in order to effectively remediate environmental PFAS contamination. Degradation pathways and mechanisms ranging from plasma reactors to photocatalytic defluorination and H+ oxidation have been explored in recent research.

### 4.1. Photo transformation

#### 4.1.1. UV photodegradation

**Fig. 5.**
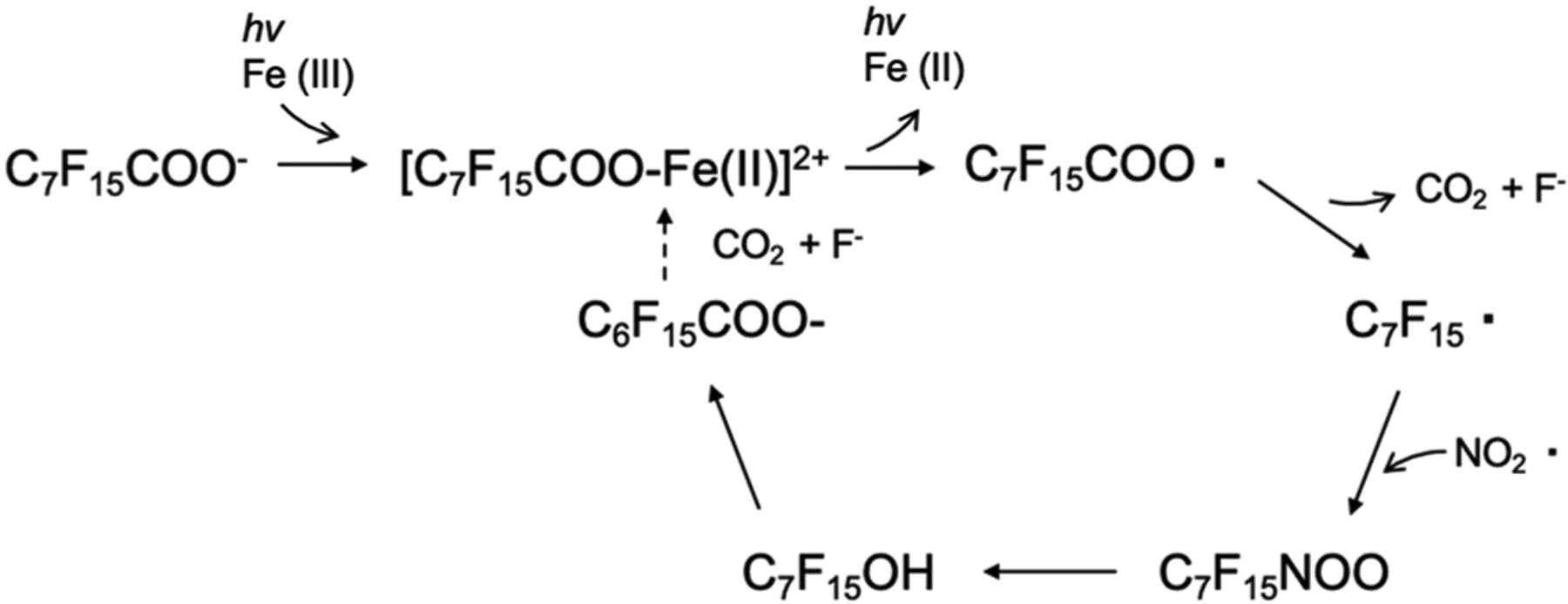
PFOA degradation scheme using ferric nitrate radicals, and the Fe^3+^/Fe^2+^ redox cycle. The cycle transforms the carboxylate acid into a radical through the iron redox reaction, followed by decarboxylation and hydrolyzed to form the fluoroalkyl alcohol which can then undergo -HF elimination. After a 12 h cycle, the PFOA is mineralized to CO_2_, and F.^−^.

UV oxidation is an advanced treatment for the degradation of organic contaminants commonly used in drinking and wastewater treatment. Mineralization of PFOA was evaluated in a laboratory scale experiment, using ferric nitrate radicals, and the Fe^3+^/Fe^2+^ redox cycle, illuminated by a 254 nm UV lamp. Yuan et al. (2020) Removal of 92% of PFOA in a 5 mg/L solution was observed after 30 min of irradiation. Calculations using DFT predicted that the degradation is based on the radical C_7_F_15_COO• formed through either ligand to metal charge transfer, or through a free radical reduction with NO_2_•. The formation of C_7_F_15_COO•, followed by decarboxylation and formation of a fluoroalkyl radical was predicted with DFT calculations supporting subsequent CF_2_ shortening (Fig. 5). After complete irradiation (12 h), the fluorine mass balance was calculated at 97% of the initial concentration (5 mg/L PFOA) as measured by ion chromatography for F^−^ ions and LC-MS for PFOA and its transformation products. Short chain PFCA transformation products were observed in the initial degradation stages supporting the DFT predictions, and decreased PFOA to 0.09% of the initial concentration (5 ppm) after 12 h. These studies are valuable as they were able to show mineralization of a very stable contaminant, while utilizing earth abundant resources like iron (III) and nitrates without the addition of a photocatalyst. Yuan et al. (2020) The lab-scale studies showed efficient degradation of PFOA, but the efficiency of this technique still need to be evaluated on a larger scale study.

6:2 Fluorotelomer sulfonate (Fig. 1) is a PFOS alternative that contain protonated sites along the fluorocarbon chain that lower the electron affinity and make them more oxidizable. Bao et al. (2021) However, these compounds degrade into the more common carboxylated PFAS. Photodegradation with an oxidative UV persulfate (UV/PS) or with reductive UV sulfite (UV/SF) systems were evaluated for 6:2 FTS removal. With the oxidative treatment, 6:2 FTS was decomposed into various shorter chain carboxylate ranging from 2 to 7 carbon PFAS in 10 min. It was observed that polyfluorinated precursors have a lower reactivity with aqueous electrons, compared to their perfluorinated counterparts. These results suggest that one treatment mechanism is not sufficient for total PFAS degradation. This technique, combined with other oxidative degradation efforts, can drive the transformation of polyfluorinated precursors to fully fluorinated compounds, which can react more readily with the aqueous electrons. Bao et al. (2021).

#### 4.1.2. Metal doped photocatalysts

Metal doped photocatalysts have also been employed to facilitate UV photodegradation of PFAS. A nanosheet photocatalyst doped with platinum nanoparticles was considered for the UV defluorination of PFOA, with methanol acting as an electron donor. Chen et al. (2021) The photocatalyst removes an electron from the methanol, which creates free radicals for reductive defluorination of PFOA. After a 12-hour exposure, the Pt/La_2_Ti_2_O_7_ nanoparticle was able to remove 50% of PFOA in an aqueous solution (50 ng/mL), with 20% of removal attributed to sorption to the photocatalyst; possible transformation products were monitored, but were not quantified. The DFT calculations displayed that the platinum doping increased charge transfer in the photodegradation reactions with PFOA. This photocatalyst has tunable band gaps that allow for the development of reactive surface sites, which can further improve catalytic reactivity. Chen et al. (2021).

A bismuth Bi/BiOI_1-x_F_x_ (x = 0.2) solid solution composed of a hollow microsphere was synthesized to optimize the bandgap and allow for absorbance of visible light while providing a high surface area. Wang et al. (2021) In 2 h of illumination, the photocatalyst was able to fully degrade 40 mg/L of PFOA. This study predicted that a superoxide anion is produced and generates the fluoroalkyl radical directly from PFOA by attacking the second carbon. Alternatively, other studies predict radical abstraction of an electron from the carboxylate group, followed by a decarboxylation step to form the fluoroalkyl radical. The fluoroalkyl radical can continue to become the perfluoro alcohols that are further defluorinated, resulting to shorter chain carboxylated PFAS. Wang et al. (2021).

### 4.2. Hole (H+) oxidation

**Fig. 6.**
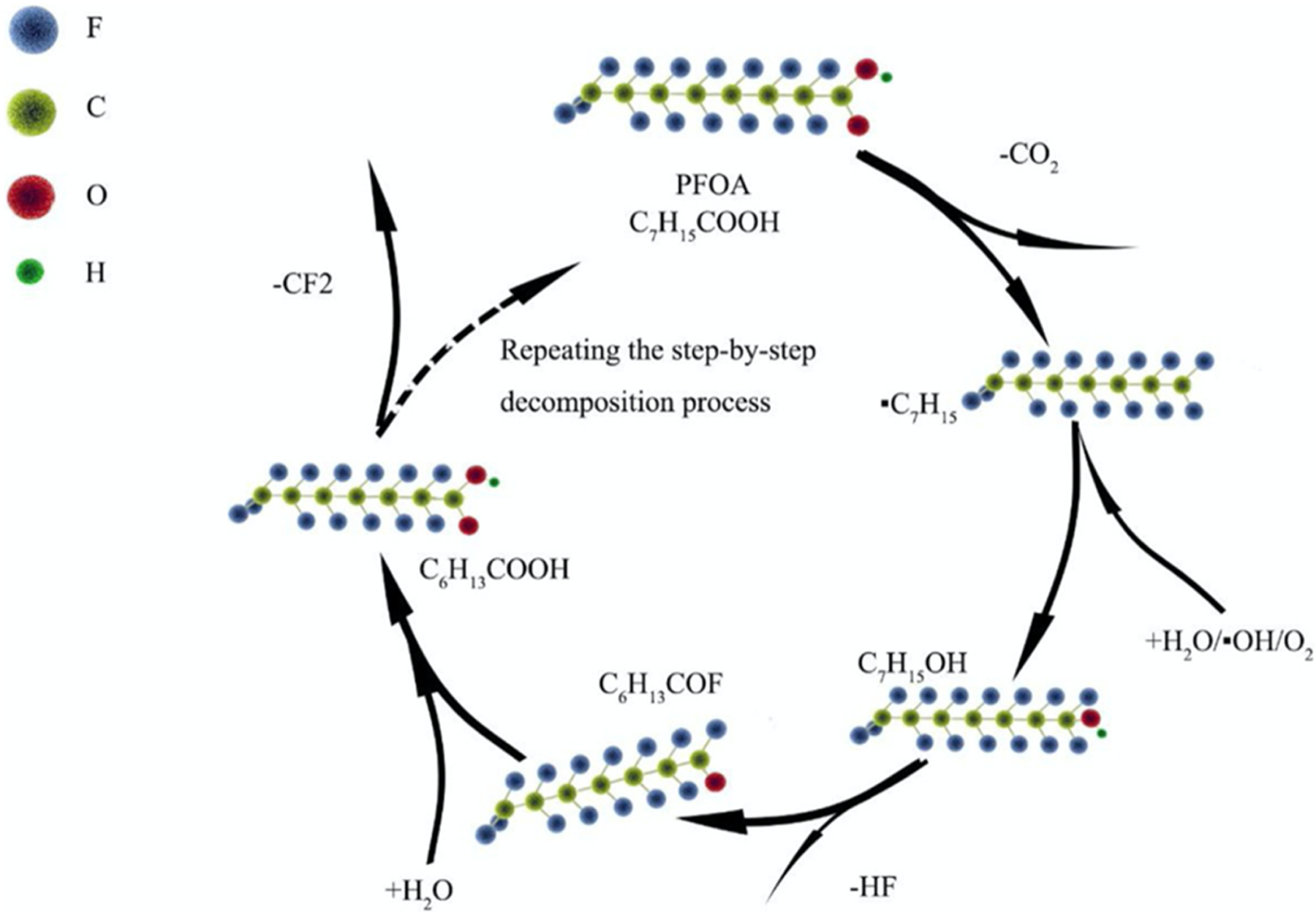
“The photocatalysts produced··OH, O_2_ and other active species, which facilitated the chain reduction of the CeF bond between C_7_F_15_COO·to form C_7_F_15_. Then,·C_7_F_15_ could react with·OH to from C_7_F_15_OH, which was subjected to the subsequent F elimination and hydrolysis and the shorter chain was formed by stepwise elimination CF_2_ units.”.

Oxidative degradation has been shown to be a strong pathway for the degradation of PFAS contaminants. The use of photocatalysts rather than electrochemical systems for efficient hole (H+) oxidation was evaluated in two recent studies. The first used oxygen vacancies along a BiOCl nanosheet as a photocatalyst with UV irradiation for the reaction. Song et al. (2019) The oxygen vacancies within the nanosheet act as an active binding site for PFOA, which are then attacked by the surplus of H+ produced within the vacancy. This photocatalyst promoted the degradation of PFOA and displayed consistent degradation after 4 reuse cycles, suggesting it can be a renewable material. However, this study did not report mechanistic understanding of the degradation, nor performed degradation rates studies to characterize the catalytic properties of the materials. A second study used BiOCl/Zn-Al hydrotalcite (B-BHZA) to catalyze the photogenerated holes that provide oxidative degradation of PFAS. The degradation pathway proposed was consistent with other oxidative degradation methods involving the decarboxylation of PFOA and formation of a fluoroalkyl radical. The fluoroalkyl radical is further hydroxylated to an alcohol, which then undergoes H-F exchange and is finally hydrated into a shorter chain carboxylate PFAS (Fig. 6). This cycle can continue to degrade the carboxylated PFAS one −CF_2_ group at a time and achieved 90% removal (0.5 mg/L) after a 6-h UV irradiation. Song et al. (2019).

### 4.3. Electrochemical remediation – oxidative and reductive

Electrochemical reactions are also used for remediation of PFAS to achieve both oxidative and reductive degradation of these contaminants. An electrochemical system was tested for the reductive degradation of PFAS using a ruthenium/nickel cathode. The cathode defluorinated and degraded PFOA with a cathode potential of − 1.25 V. Zhu et al. (2022) Because of the empty D-orbitals on Rh, the cathode has a strong affinity for halogens, such as fluorine. Dehydrofluorination occurs as Rh interacts with fluorine molecules, elongating and weakening the C-F bond and subsequent reduction by active atomic hydrogen. This system allows for degradation of PFOA under relatively mild conditions, where H-F exchange along the alkyl chain followed by cleavage of the exchanged portion of the molecule results in short chain carboxylated PFAS that are then further decomposed. Zhu et al. (2022) This study did not report the rates of degradation for PFAS, which is crucial information for the widescale implementation of the techniques.

Photoelectrochemical (PEC) systems with graphene oxide titanium dioxide anode that is coated on a fluorine doped tin oxide (GOP25/FTO) glass to degrade PFOA and PFOS were evaluated. The combination of graphene oxide as a support with the titanium dioxide reduced the band gap of the anode, resulting in improved efficiency of the PFOA decomposition. Yang et al. (2020) This technique yielded 92.8% removal of PFOA from water (0.5 mg/L) in 4 h, with the primary transformation pathway through decarboxylation and oxidation. Defluorination, hydroxylation, and chlorine substitution were observed as well, though with lower yields than observed with the decarboxylation pathway. These studies used NaCl as electrolyte, showing that an abundance of chlorine ions in the sample matrix can result into new transformation products during the PEC degradation process. PFOS was similarly degraded, with the loss of the sulfate head group followed by the same decarboxylation pathway. Further, PFOS (0.5 mg/L) underwent defluorination, hydroxylation, and chlorine substitution and was degraded completely into various shorter chain intermediates within 4 h. Yang et al. (2020).

A boron doped diamond electrode was used for oxidative degradation of PFOA and PFOS in leachate water samples. Pierpaoli et al. (2021) The boron electrode provided a higher current density (75 mA/cm compared to the GOP25/FTO (16.7 mA/cm (Ahrens et al., 2011)). At this current, the electrode removed an average of 78% and 80% of PFOS and PFOA contamination in 8 h, respectively, while 41% and 45% removal was observed after 8 h with a 25 mA/cm (Ahrens et al., 2011) current. This suggests that boron doped diamond electrodes can degrade PFOA and PFOS in water samples, but the rates of PFAS degradation in comparison to other electrochemical systems has not been considered. Pierpaoli et al. (2021).

### 4.4. Plasma reactions for PFAS degradation

Plasma reactors appear to be effective for degradation of PFAS contaminants in the enviroment. Zhan et al. (2020) Soil contaminated with PFOA was treated using plasma corona discharge (30 kV). Short chain transformation products made up 51% of the mass balance, while 19% was mineralized and 1% volatilized. DFT calculations indicate that the carbon-carbon bond breakage leads to the subsequent loss of −CF_2_ units. Additionally, the promotion of *Planctomycetes* and *Nitrospira* indicate that plasma reactions can enhance the nitrogen cycle. Zhan et al. (2020) A bench-scale plasma degradation experiment was also performed in landfill leachate to degrade both PFOA and PFOS. Singh et al. (2021) After a 10 min reaction, 90% of the PFOA and PFOS contamination were removed. The greatest removal efficiency achieved with this reaction was 99.9% removal of the long chain PFAS, 44–99.99% removal of legacy PFAS, and 10–99.9% removal of short chain PFAS after 75 min of plasma treatment. The reaction additionally reduced the total organic carbon (TOC) by 34%. The addition of cetrimonium bromide, electrostatic interactions between the cationic surfactant and PFAS improved the removal of short chain PFAS considerably (PFBS (95%), PFPeA (45%), PFBA (42%), PFHxA (39%)). It was predicted in this study that the organic matter in the leachate was more readily degraded in the plasma than the short chain molecules, suggesting that much longer reaction times is needed to effectively degrade PFAS in these environments. Singh et al. (2021) Plasma reactors though are known for their high energy requirements and high operational cost for remediation, and therefore are not widely accessible for PFAS remediation.

A dielectric barrier discharge plasma was tested for the degradation of C6/C6 perfluorohexyl bisphosphinic acid (C6/C6 PFPiA) (Fig. 1), a PFAS alternative used as a pesticide defoaming agent and emulsifier; C6/C6 PFPiA was widely detected in waste and surface waters. Gao et al. (2022) This study evaluated the removal efficiency, effect of pH, and identity of transformation products of the reaction using LC-high resolution mass spectrometry, while the mechanisms for degradation were modeled using DFT calculations. It was observed that the removal efficiency was directly related to the applied voltage, with 94.5% removal achieved with 18 kV discharge voltage (15 min, pH 7.5). Additionally, the removal efficiency was improved to 98.9% using the same parameters, but at a pH of 11.5. The degradation with this method showed first order rate kinetics with k_pH11.5_ = 0.34 min^−1^. Through monitoring ROS including •OH, O_2_•^−^, and O_2_, it was observed that ROS play a significant role in PFAS degradation as the three ROS concentrations decreased with the addition of C6/C6 PFPiA. These reactions lead to the cleavage of the carbon phosphorous bond, followed by subsequent cleavage of −CF_2_ groups as a result of electron transfer and free radical reactions. These observations were supported by the detected transformation products, including various short chain PFCAs, F^−^, CH_3_COO^−^ and ${\text{C}}_{2}{\text{O}}_{4}^{2-}$, as well as through DFT calculations. Gao et al. (2022).

### 4.5. Metallic structures for PFAS degradation

Studies on reductive transformation with metal catalysts mounted on carbon supports for the degradation of PFOS and PFOA were performed. In one study, nickel and iron nanoparticles supported on activated carbon (nNiFe°-AC particles) were explored. These metals are used because zero valent iron degrades PFAS readily, while nickel is a low cost, highly reactive catalyst with multiple oxidation states. Zenobio et al. (2020) Treatment using these nanoparticles resulted in a 50% decrease in PFOS over 24 h, followed by a plateau which suggested that no further degradation was occurring. After degradation, 97% of the F^−^ ions produced were accounted for by ion chromatography analysis. The nNiFe°-AC particles are good candidates for remediation in wastewater treatment facilities as permeable reactive barriers because the technique showed no pH dependency. Additionally, very few degradation intermediates were observed, suggesting a low risk of secondary PFAS contamination from PFAS transformation products. Zenobio et al. (2020).

Another study observed significant adsorption and degradation of PFOA in wastewater as a result of bimetallic carbon structure, C_Ni_-Al_2_O_3_ structure (AlC_Ni_), with mechanochemical stirring. Liu et al. (2021) After 3 h, 98% of PFOA was remediated, with the produced F^−^ ions mass balance suggesting 70% mineralization of PFOA. The common pathway of defluorination, hydroxylation, and carboxylation were again observed for the degradation of PFOA. The catalyst preformed more readily at lower temperature due to the dehydration of the AlC_Ni_ catalyst. Liu et al. (2021) This technique is a simple method without the need of additional chemicals and can be widely applied for the reduction of PFOA.

## 5. Biological treatment

Bioremediation involves biological treatment using naturally occurring organisms for the degradation and removal of contaminants in the environment. This includes microbial degradation in soils and sludge, as well as the use of crops for sequestration of chemicals. Additionally, synergistic interactions with biological and abiotic materials, such as enzymes paired with photocatalysts, have been designed to break down persistent chemicals.

### 5.1. Sequestration into constructed floating wetlands (CFWs)

Physical treatment of PFAS includes the capturing of chemicals for the purpose of physical separation from the environment. With constructed floating wetlands (CFW), the concept of using biological materials, like Phragmites australis and other common crops, for sequestration of long chain PFAS was evaluated using contaminated storm water (0.2 – 30 μg/L) over a 28-day exposure. Awad et al. (2022) The study observed higher accumulation of PFOS than PFOA in the roots, while the opposite was observed in the plant shoots. Notably, the uptake of PFAS into the CFW decreased as the concentration of PFAS increased, suggesting that it can be effective for PFAS removal from contaminated waters. Awad et al. (2022) Higher levels of contamination would still be better addressed using a different treatment technique, such as photocatalysts or electrochemical techniques discussed above.

### 5.2. Biodegradation pathways

Biodegradation of PFOA is rare, but enzyme catalyzed oxidative humification reactions have been observe to lead to PFOA degradation in soil slurry (40% degraded after 140 days) and water (24% degraded after 36 days). Luo et al. (2018) Free radical chain reactions from the laccase soybean meal produced free radicals that can attack the carbon-carbon bonds of the PFOA molecule, which was identified to be the primary degradation pathway in soil samples. This was determined based on the various, partially fluorinated, degradation products that were observed following the reactions. The study displayed that soybean meal can provide free radicals to effect the breakdown of PFOA into various partially fluorinated byproducts within the environment. Luo et al. (2018).

6:2 Fluorotelomer sulfate (6:2 FTS) is a polyfluorinated PFAS known to bio-transform in the environment, but the pathways of degradation are not well known. Yang et al. (2022) To gain a better understanding of the pathway, the gene expression of *Rhodococcus jostii* RHA was monitored; a bacteria capable of degrading 6:2 FTS due to the presence of oxygenases, dehalogenases, and desulfonating enzymes. It was observed that the sulfonate head group hindered defluorination, since the 6:2 fluorotelomer alcohol (Fig. 1) was readily defluorinated under the same processes. In the absence of sulfur media, alkanesulfonate mono-oxygenase was capable of desulfonating the 6:2 FTS. This desulfonation facilitated the subsequent defluorination by alkane mono- oxygenase, haloacid dehalogenase, and cytochrome P450 enzymes. Yang et al. (2022) With a better grasp on biodegradation pathways, future degradation mechanisms can be designed to include these enzymes for degradation, or using synthesized materials to mimic the enzymatic degradation, as well as design synergistic systems to improve the overall degradation of PFAS as a whole.

### 5.3. Synergistic degradation – biotic and abiotic

Efforts to achieve PFAS degradation through the combined use of biotic and abiotic treatment strategies have been evaluated in recent studies. Microbial degradation was paired with adsorption and photocatalytic degradation for treatment of PFOA at low concentrations (500 μg/L). Ding et al. (2021) This technique used a bismuth oxyhalides cathode (Bi_12_O_17_Cl_2)_ where the PFOA is decarboxylated through a H+ electron abstraction, followed by defluorination with hydroxide radicals produced by the photocatalyst. The addition of microbes to promote biodegradation of the decarboxylated PFAS resulted in the formation of defluorinated transformations products. This synergistic technique provided considerable improvement in the degradation of PFOA (30.7% increase) compared to the use of the photocatalyst alone. The subsequent cleavage of −CF_2_ from the PFOA was observed with this synergistic system that harnesses natural processes for the degradation of PFOA, with the additional benefit that it lowered the TOC (44.2% removed). Ding et al. (2021).

The degradation of PFAS using a “micro bioreactor” with functionalized iron nanoparticles on living diatoms has also been reported. Albert et al. (2020) The strategy employs the Fenton reaction for the production of hydroxyl, as well as the surplus of superoxide and hydroxyl radicals produced by diatoms under stressed exposure conditions. It was observed that hydroxylation and oxidation were two major pathways for the defluorination of PFOA and PFOS, achieving 95% and 90% removal (24 h) at pH 9, respectively. These functionalized diatoms were readily recycled for further PFAS degradation, and showed no signs of cytotoxicity over an extended period of time, making this an ideal, green approach for PFAS remediation. Albert et al. (2020) Large scale evaulation of these synergistic biotic and abiotic degradation efforts should be performed under real-world conditions to assess their practical applications.

## 6. Fate of PFAS in the environment

Once released into the environment, PFAS will hardly breakdown and will persist in the terrestrial or aquatic environment. The fate and transport of PFAS in the environment will depend on their chain lengths, the types of head group, presence of ether bonds, ionic state, the environmental pH, and the extent of fluorination in the carbon chain. These properties will influence the sorption, bioaccumulation, volatilization, and potential for degradation and treatment that can be expected to occur for PFAS. Understanding the fate of PFAS in various environmental compartments can provide insight into the mechanisms of degradation and persistence of PFAS in the natural environment.

### 6.1. Removal in drinking water treatment facilities

A notable publication evaluated the sorption and removal of 14 PFAS (n = 14) throughout commonly used drinking water processes. Kim et al. (2020) Five drinking water treatment plants (DWTPs) were evaluated, all of which utilize sedimentation and coagulation, followed by chlorination, advanced treatment processes such as ozonation. It was observed that the removal efficiencies were directly related to the chain length of the PFAS. Due to the increased electrostatic affinity, sulfonated PFAS had stronger removal efficiencies (21.4% greater) relative to the carboxylated PFAS of the same chain length. It is important to note that the long-chain and sulfonated PFAS were removed by the GAC (~40%), while the shorter chain and carboxylated PFAS had low or negative removal efficiencies (<10%). Kim et al. (2020) Knowledge on the possible breakthrough and low removal efficiencies is important since short chain PFAS are known to be formed during degradation of long chain PFAS. Designing and evaluating new removal technologies that will eliminate a wide range of PFAS, particularly the short chain molecules, is a crucial direction for future research to help mitigate the PFAS crisis.

### 6.2. Sorption and degradation in waste water and sewage sludge

PFAS have been detected in wastewater, groundwater, surface water, source and drinking water samples globally. Guardian et al. (2020); Zacs and Bartkevics (2016); Seo et al. (2019) Understanding the fate of PFAS removal using current water treatment technologies is crucial for the development of newer and effective clean-up techniques. The sorption capacity of PFOA was evaluated in aerobic, anaerobic, and enhanced biological phosphorous removal sewage sludge to better understand the behavior of PFAS molecules throughout the waste water treatment plants (WWTPs). Yan et al. (2021) Additionally, they evaluated the effect of extracellular polymeric substances (EPS) for the adsorption and removal efficiencies. The highest adsorption was observed in EPS, through the mechanism of protein binding; the C-F chain interacts with the aromatic portion of the protein, while the carboxylate and the protein amide interact electrostatically. Yan et al. (2021) This study is helpful for the subsequent removal of PFOA from WWTPs, but does not elucidate interactions of other PFAS with sewage sludge. Additionally, these treatment techniques were designed for pharmaceuticals and other pollutants, and at this point the understanding of PFAS removal in DWTPs is not widely understood beyond what is discussed above.

A slurry of biosolids were collected and incubated (150 d) with *Acidimicrobium sp*. strain A6, with and without the addition of ferrihydrite. Huang et al. (2021) These were fortified with PFOA to concentrations of 0.2 and 10 mg/L, and incubated for 150 d. Autoclaved controls were also treated with 10 mg/L of PFOA. Samples were taken at 30-day increments, and 506 mg/L of 6-line ferrihydrite was added on day 90. Shorter chain PFCAs, F-, and a decrease in the overall PFOA concentrations were observed. None of the controls resulted in the defluorination or decomposition of PFOA, which suggests the decomposition is a result of the *Acidimicrobium sp*. strain A6 activity, though a mechanism for degradation was not proposed by the study. The health of the microbial community was significantly impacted by the high concentrations of PFAS. Huang et al. (2021).

### 6.3. Soil and sediment stabilization

Stabilization and solidification (s/s) is a technique used in contaminated soils, where binders and additives are added to a contaminated area for both physical and chemical stabilization. Sorengard et al. (2019) The leaching and stabilization of PFAS contamination were evaluated using various s/s additives in a lab-scale study. Additives with GAC, such as powdered activated carbon and Rembind^®^, provided significant reduction (P < 0.05) in leaching for PFPeA, PFHxA, PFHpA, PFOA, PFNA, PFDA, PFUnDA, PFBS, PFHxS, PFOS, 6:2 FTSA, 8:2 FTSA, and FOSA compounds. The shorter chain molecules (PFHxA, PFHpA, and the PFBS) have lower stabilization efficiencies using the GAC additives than the long chain PFAS, which is consistent across literature to date. Rembind^®^ decreased the stability of the soil structure by 38% but remained within the unconfined compressive strength required by the USEPA. The GAC additives provided effective remediation through s/s without significant loss in stabilization, but the study should be repeated at the field scale. Sorengard et al. (2019).

A study by Sörengård examined the efficiency of s/s techniques for reducing the PFAS contamination in leachate using a pilot-scale simulation. Sorengard et al. (2021) It was observed that the addition of GAC to the s/s formulation had a marked impact on removal efficiency. PFAS with > 6 chain lengths had 98% removal after 6 years of simulated rain water on 3 tons of AFFF contaminated soils, and achieved levels of PFAS close to ground (Swedish guideline value 0.045 μg L^−1^) and drinking (Swedish guideline value 0.090 μg L^−1^) water advisory levels. This study also observed two previously undiscovered PFAS through suspect screening using the *NORMAN Digital Samples Freezing Platform*: 3:2 FTOH and heptafluoropiperidin butane. Laboratory scale experiments show comparable results to the pilot scale study, but the pilot scale results showed removal magnitudes larger than what was observed in laboratory samples. Sorengard et al. (2021).

The use of GAC for the stabilization of PFAS in subsurface environments has been shown to be effective for long chain PFAS (>6 carbon chain), but for shorter chain PFAS these materials are not effective. An anion exchange resin, Amberlite^®^ IRA910, was tested as an injectable absorption barrier to improve stabilization of short chain PFAS in groundwater using batch reactors and column studies. Liu et al. (2022) Efficient binding of PFHpA, PFBS, PFOA, PFHxS, PFNA, and PFOS was observed, ranging from 30.51% to 96.44% removal efficiency at 10 mg/L. The hydrophobicity of the PFAS molecule was directly related to the removal efficiency of the sorbent, and no significant competitive binding with the organic matter was observed. This study highlights the effects of the organic matter specific to each real-application site, and the authors encouraged future studies to evaluate the long-term effectiveness of the sorbent. Liu et al. (2022).

### 6.4. Clay mineral photo-transformation

Photo-transformation in clay minerals of PFOSA, a sulfonamide precursor to the more stable carboxylated PFAS, was evaluated and compared with photo-transformation rates in aqueous solutions in the presence of phosphate buffer (pH 7.2). Lv et al. (2020) In the presence of montmorillonite, a natural clay mineral, the degradation to PFOA to the shorter chain carboxylates was facilitated by reacting with superoxide anions and hydroxide radicals. These radicals were also utilized with synthesized oxidative degradation techniques discussed earlier in the review. The radical reactions lead to short chain carboxylated transformation products from free radical reactions and rearrangements. The degradation pathways of PFOSA to PFOA and shorter chain PFAS are well known, but the assisted degradation by montmorillonite suggests that this degradation can occur in the environment. This transformation pathway is similar to the degradation of other PFAS, so it is predicted that fluorotelomers and phosphate PFAS could also be degraded by the clay minerals. Lv et al. (2020) However, the reaction could be a potential source of novel short chain PFAS contaminants (secondary contamination) in the environment. The mechanisms observed in this study are consistent with other observed degradation pathways of FOSA and PFCA discussed in this review. Also, it is important to note that these degradation pathways can be a route of secondary contamination of PFCA within the environment.

## 7. Conclusion and research needs

PFAS distribution, toxicity, and removal and remediation techniques continue to be widely studied in the environment, and therefore information on these contaminants that are known as “forever chemicals” are increasingly published in the scientific literature. This review highlights some of the more recent findings in PFAS toxicity, removal, and remediation published in *Journal of Hazardous Materials* to facilitate the spread of knowledge, as well as inspire and inform future works. Toxicity studies using computational modeling, plant, animal, and human subjects show the risk associated with various levels of exposure. Additionally, evidence are presented that shows different classes and chain lengths of PFAS can induce different impacts on humans, animals, and the environment. It was observed that PFAS exposure can impact the regulation of stress and immune responses, hormone production, as well cause various oxidative stress reactions, consistent with earlier toxicological studies in mice and rat toxicity studies. The publications highlighted in this review also evaluated pathways and mechanisms of toxicity at a cellular and genomic levels in animal models, which can be used to inform human toxicity studies.

Removal techniques for the sequestration of PFAS from environmental water samples were discussed using C18, WAX and liquid-liquid extraction techniques. The techniques discussed here were able to achieve up to 95% PFAS removal within contaminated systems, but had high variability in removal efficiencies reported between legacy and alternative PFAS. Though removal techniques can separate these contaminants from the environment, they are only a piece of the larger solution. Physical removal techniques do not address the need to destroy the contaminants, and results in an abundance of material waste containing high concentrations of PFAS. Destruction techniques can be used to complement these removal strategies, and completely remediate PFAS from the environment. These techniques often follow known decarboxylation, desulfonation, and/or defluorination pathways using electrochemical, photocatalytic, microbial and redox reactions. It was shown in these studies that degradation techniques for PFAS are not universally effective for all the PFAS classes, and that remediation efforts must be catered to the specific contaminants of interest. For example, perfluorinated molecules were more readily degraded than their polyfluorinated precursors with aqueous electrons, and therefore degradation with this method would be slow for many polyfluorinated PFAS precursors. Different approaches for degradation can be combined to produce the most efficient degradation processes to cover a larger range of PFAS concentrations and classes. Synergistic approaches which combine different routes of degradation, as well as chemical and biological degradation techniques have become more common due to their unique ability to destroy more PFAS at both low and high concentrations, and their efficiencies should be evaluated at environmentally relevant scales. The research articles reviewed in this paper will be made *open-access* until May 2023 in order to further advance the understanding of the impacts of PFAS, in hope that it will aid global efforts to provide PFAS-free food and water to all. Moving forward, it is crucial that more PFAS classes be evaluated for their ecological, animal, and human toxicities, as well as the efficiency of the removal and remediation techniques. These are important next steps since PFAS alternatives are designed with different chemical properties, and might not react to removal and remediation efforts in the same manner as the legacy PFAS, PFCA and PFSA. Promising techniques discussed here, such as the mineralization of PFOA with ferric nitrate radicals, and the Fe^3+^/Fe^2+^ redox cycle, should be further explored and validated for real-world, large-scale, remediation efforts within the environment.

## HIGHLIGHTS

- A review of recent research on PFAS toxicity, removal, and remediation.
- PFAS exposure linked to dysregulation of stress, immune, and hormonal responses.
- Degradation techniques for PFAS are not universally effective for all the PFAS classes.
- Synergistic degradation techniques can be designed specific to classes of PFAS.
- Environmental models can be used to design anthropogenic PFAS degradation techniques.

## Environmental implication

This manuscript highlights key research which focuses on the impacts of PFAS exposure, as well as the solutions to environmental PFAS contamination through sequestration and removal. The fate of PFAS in the environment as well as current treatment processes is reviewed and can provide insight into key degradation pathways of the forever chemicals. The articles reviewed here will be made open access in order to facilitate the distribution of research on a major environmental contaminant.
